# Effects of wall materials on the physicochemical properties of spray-dried bitter gourd (*Momordica charantia* L.) powders

**DOI:** 10.1038/s41538-024-00278-7

**Published:** 2024-06-20

**Authors:** Yuanyuan Deng, Guang Liu, Huimin Zhang, Pengfei Zhou, Xiaojun Tang, Ping Li, Zhihao Zhao, Yan Zhang, Zhangying Wang, Zhencheng Wei, Mingwei Zhang

**Affiliations:** 1 Sericultural & Agri-Food Research Institute, Guangdong Academy of Agricultural Sciences / Key Laboratory of Functional Foods, Ministry of Agriculture and Rural Affairs / Guangdong Key Laboratory of Agricultural Products Processing, Guangzhou, 510610 China; 2 Crops Research Institute, Guangdong Academy of Agricultural Sciences / Key Laboratory of Crop Genetic Improvement of Guangdong Province, Guangzhou, 510640 China

**Keywords:** Agriculture, Plant sciences

## Abstract

Bitter gourd has numerous health-promoting effects on the human body. However, its use has been greatly limited due to its poor acceptance by consumers, resulting from its strong bitterness. This study investigated the effects of five wall materials, namely, soybean protein isolate, gum arabic, maltodextrin, resistant starch, and a soybean lecithin calcium caseinate mixture, on the physicochemical properties of spray-dried bitter gourd powders. The results showed that all five wall materials reduced the moisture content, water activity, browning degree, agglomeration, and bitterness of the spray-dried bitter gourd powder. Maltodextrin was found to be the most effective at reducing water activity, while soybean protein isolate was best at protecting the colour, and the soybean lecithin calcium caseinate mixture was best at reducing hygroscopicity and masking bitterness. Additionally, all five wall materials improved the preservation of flavonoids, saponins, and vitamin C, with soybean protein isolate being the most effective in improving the total flavonoid retention ratio and the soybean lecithin calcium caseinate mixture being the best in improving the retention ratios of total saponins and vitamin C. The spray-dried bitter gourd powder prepared with soybean protein isolate had the highest antioxidant activity and α-glucosidase inhibitory activity. These results are significant for understanding the relationship between wall materials and the physicochemical properties of spray-dried powder. Additionally, these materials provide bitter gourd product manufacturers with useful guidance for producing high-quality products. Furthermore, the results could provide useful insights for processing fruits with similar product characteristics, thus contributing to the enrichment of food processing knowledge.

## Introduction

In traditional Chinese medicine, dried or fresh bitter gourd is commonly used to treat heat stroke, fever, dysentery, stomach problems, inflammation, and other disease symptoms via decoction^1–4^. Modern medical research has also shown that bitter gourd has functions such as lowering blood sugar, inhibiting inflammation, eliminating free radicals in the body, combating cancer, treating malaria and AIDS, and regulating immunity^5^. Nutritional analysis has shown that bitter gourd is rich in carbohydrates, protein, dietary fibre, vitamins, and minerals^6–8^. Additionally, it contains a variety of active plant compounds, such as saponins, polysaccharides, polyphenols, flavonoids, and essential oils^5,9^, indicating that it may possess important bioactive functions for humans.

However, many active compounds in bitter gourd present a bitter taste^10^. Four triterpenoid saponins with a bitter taste, namely, momordicine I, momordicoside II, momordicoside K, and momordicoside L, have been identified in bitter gourd^11–14^. Because of the unpleasant taste and short shelf life of fresh bitter gourd, the consumption of bitter gourd is limited. To improve the health-promoting benefits of bitter gourd and for easy consumption, various bitter gourd products, such as bitter gourd tea^15,16^, bitter gourd solid drink^17^, bitter gourd and mulberry leaf biscuits^18^, bitter gourd buckwheat cake^19^, and bitter gourd effervescent tablets^20^, have been continuously developed. However, currently, available bitter gourd products on the market are still not widely accepted by consumers because the bitter taste is still not satisfactorily attenuated or masked^21–23^.

The development of most bitter gourd products usually involves drying. Spray drying is a common drying method used in the food industry and can also be used to encapsulate certain substances^24–26^. The most commonly used wall materials for spray drying are starch and its derivatives, lipids, and proteins. Among them, gum arabic (GA)^27–29^, soybean protein isolate (SPI)^30–32^, maltodextrin (MD)^29,33–35^, sodium alginate^36,37^, whey protein^35,38^, and soybean lecithin calcium caseinate mixture (LCC)^39^ have been found to have the ability to mask bitterness and improve other physicochemical properties in spray-dried products. Ferraz et al. discovered that whey protein isolate, MD, and their combinations can protect active compounds during storage in spray-dried paprika and cinnamon oleoresin products^35^. Mishra et al. reported that spray-dried amla juice powder made with MD had less hygroscopicity, an acceptable colour and potent free radical scavenging activity^34^. Ma et al. discovered that spray drying with whey protein concentrate as a wall material was beneficial for reducing the bitter taste and hygroscopicity of whey protein hydrolysate^40^. Subtil et al. reported that GA was able to attenuate the bitter taste of hydrolysed casein in spray-dried products^27^. Tan et al. reported that MD and GA encapsulated aqueous bitter melon extract and effectively protected bioactive compounds from degradation during the spray drying process^41^.

However, to date, there has been a lack of systematic research on the ability of different wall materials to mask bitterness and the retention of functional components. Additionally, the types of wall materials used for encapsulation in the spray drying of bitter gourd are relatively limited.

The purpose of this research was to identify the most suitable wall materials for producing spray-dried bitter gourd powders with good application potential by systematically comparing the bitterness-masking abilities of different wall materials and their effects on the physicochemical properties of spray-dried bitter gourd powders. Furthermore, this research aims to lay the foundation for developing a spray-drying bitterness encapsulation technology that can reduce the bitterness of bitter gourd powders and improve the retention ratios of active substances.

## Results

### Effects of wall materials on the moisture content and water activity of spray-dried bitter gourd powders

Table 1 shows the effects of the wall materials on the moisture content and water activity of the SD-BGP. The moisture content and water activity of the NE SD-BGP were the highest, at 9.67 and 0.32%, respectively. However, the moisture content of the SD-BGP significantly decreased after the application of the five wall materials in the spray-drying process. The descending order of the moisture content of the SD-BGP after encapsulation with the wall materials was RS, LCC, SPI, MD and GA. The SD-BGP produced with protein-based wall materials (SPI, LCC) had a higher moisture content than the samples produced with GA and MD. This might be attributed to the hard shells formed from the protein-based wall material of higher viscosity, which limited the diffusion of water from the interior of the particles to the particle surface, as noted by ref. ^42^. Similarly, the application of the five wall materials in the spray-drying process resulted in a significant decrease in the water activity of the SD-BGP compared with that of the NE SD-BGP. The descending order of the water activity of the SD-BGP after encapsulation with the wall materials was LCC, SPI, RS, GA and MD.

**Table 1:** Moisture contents of the bitter gourd powders prepared using spray drying with different wall materials ($\bar{{\rm{\chi }}}$ ± SD)

Wall materials	Moisture content (%)	Water activity
NE	9.67 ± 0.18d	0.32 ± 0.01d
SPI	5.07 ± 0.25b	0.25 ± 0.01c
LCC	5.83 ± 0.21b	0.27 ± 0.00c
GA	4.01 ± 0.16a	0.22 ± 0.01b
MD	4.10 ± 0.16a	0.21 ± 0.01a
RS	7.13 ± 0.19c	0.25 ± 0.00c

Different lowercase letters in a column indicate significant differences (*p* < 0.05).

*NE* nonencapsulated, *SPI* soybean protein isolate, *LCC* lecithin calcium caseinate, *GA* gum arabic, *MD* maltodextrin, *RS* resistant starch.

### Effects of wall materials on the solubility of spray-dried bitter gourd powders

Table 2 displays the WSI and dispersion time of the SD-BGP prepared using different wall materials. The data revealed that the NE SD-BGP had the lowest solubility, with a WSI of 37.6 ± 0.03%. In contrast, the WSIs of the SD-BGPs prepared with the five wall materials ranged from 40.5 ± 0.01% to 84.8 ± 0.02%, indicating that the application of these five wall materials significantly improved the solubility of the SD-BGPs. Among these, the SD-BGPs prepared with GA and MD exhibited the highest solubility, with a WSI of over 80%, whereas those prepared with the remaining three wall materials had substantially lower solubilities, with a WSI below 50%. The highest WSI for MD-encapsulated powder observed in this study is consistent with the findings of Mishra et al., who reported a high WSI for spray-dried amla powder prepared with MD^34^. The possible reason that the SD-BGP performed well in terms of WSI in the cases of GA and MD is that both wall materials are highly soluble in water^27,34,43^.

**Table 2:** Solubility of the bitter gourd powders prepared using the spray-drying technique with different wall materials ($\bar{{\rm{\chi }}}$ ± SD)

Wall materials	WSI (g/100 g DW)	Dispersion time (s)
NE	37.6 ± 0.03a	2.53 ± 0.22a
SPI	40.5 ± 0.01b	4.89 ± 0.19a
LCC	42.3 ± 0.00b	5.82 ± 0.34a
GA	80.5 ± 0.01c	256 ± 11.44c
MD	84.8 ± 0.02d	55.43 ± 1.03b
RS	42.6 ± 0.01b	1.20 ± 0.16a

Different lowercase letters in a column indicate significant differences (*p* < 0.05).

*NE* nonencapsulated, *SPI* soybean protein isolate, *LCC* lecithin calcium caseinate, *GA* gum arabic, *MD* maltodextrin, *RS* resistant starch.

Table 2 also shows that the dispersion of the NE SD-BGP was satisfactory, with a dispersion time of 2.53 ± 0.22 s. However, the performances of the SD-BGPs prepared with the five different wall materials varied greatly, with dispersion times ranging from 1.20 ± 0.16 s to 256 ± 11.44 s. Among them, the SD-BGPs prepared with SPI, LCC, and RS performed well, with dispersion times not significantly different from those of the NE SD-BGP. However, the SD-BGPs prepared with MD and GA were difficult to disperse, with dispersion times of 55.4 and 256 s, respectively, which are both significantly greater than those prepared with SPI, LCC, and RS. The SD-BGP encapsulated with GA had the longest dispersion time (256 ± 11.44 s), which may be due to its small particles and low moisture content. The negative effect of MD on powder dispersion discovered in this study was consistent with the findings of Baldelli et al., who reported that an increase in MD as a wall material caused a decrease in dispersibility in water in milk powders^44^.

### Effects of wall materials on the colour of spray-dried bitter gourd powders

**Fig. 1:**
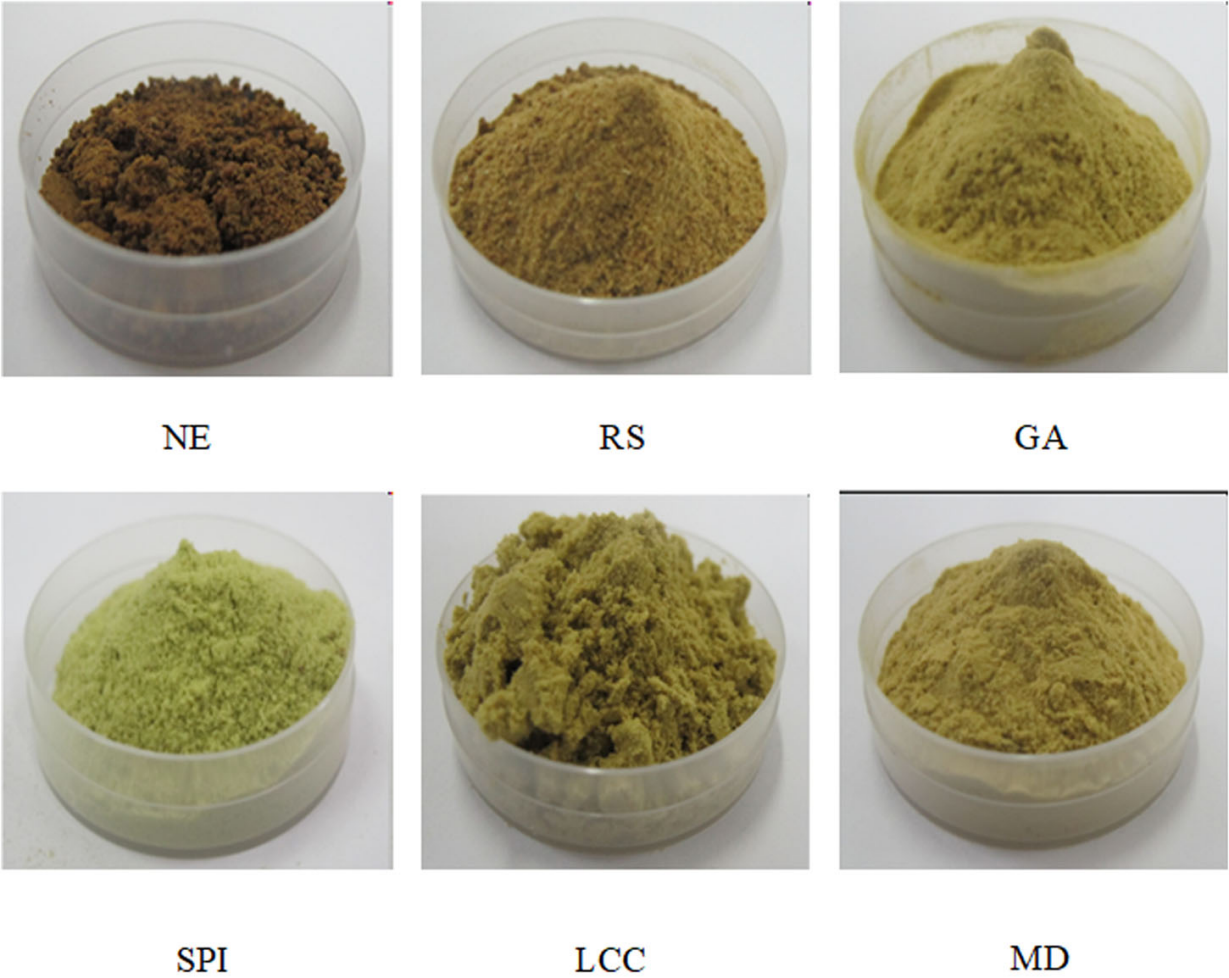
Dried bitter gourd powders prepared with different wall materials. NE nonencapsulated, SPI soybean protein isolate, LCC lecithin calcium caseinate, GA gum arabic, MD maltodextrin, and RS resistant starch.

Colour is a crucial indicator of the quality of a powder, and it is closely related to its organoleptic properties. The colour of bitter gourd powder mainly depends on the spray-drying conditions and wall materials used. The colour parameters of the bitter gourd powders, including L*, a*, b*, chroma and hue angle, are presented in Fig. 1 and Table 3. The results show that the NE SD-BGP was reddish-brown (0° < H° < 90°), with a relatively low yellow value and brightness value, a high red value, and a low chroma. The addition of the five wall materials significantly decreased browning and increased the green value, yellow value, brightness value, and chroma. Except for the resistant starch, the SD-BGPs prepared with the other four wall materials were yellowish-green, with some green colour retained. In general, protein-based wall materials, especially SPI, were excellent at preserving the colour of the SD-BGP. The possible reason for the better preservation of the green colour in the SD-BGP prepared with protein-based wall materials is that the green colour is better protected due to the rapid migration of proteins to the particle surface and quick formation of films during the spray-drying process^38,45^. The colour differences between the SD-BGPs prepared with SPI and those prepared with LCC may be because the colour of the SD-BGPs prepared with LCC was affected by the colour of the lecithin in the LCC mixture.

**Table 3:** Effects of wall materials on the colour of the spray-dried bitter gourd powders

Wall materials	Colour parameters ($\bar{{\rm{\chi }}}$ ±SD)				
	L*	a*	b*	Chroma	Hue angle
NE	49.713 ± 0.39a	7.18 ± 0.14f	18.88 ± 0.17a	20.20	69.19
SPI	67.13 ± 0.24e	7.75 ± 0.06°	30.71 ± 0.13d	31.67	−75.84
LCC	66.54 ± 0.09d	3.68 ± 0.02b	30.85 ± 0.1d	31.07	−83.19
GA	65.77 ± 0.07c	2.61 ± 0.13c	30.98 ± 0.14d	31.09	−85.19
MD	67.36 ± 0.16e	0.31 ± 0.03d	29.3 ± 0.16c	29.31	−89.4
RS	62.73 ± 0.13b	0.24 ± 0.0e	25.72 ± 0.14b	25.72	89.47

Different lowercase letters in a column indicate significant differences (*p* < 0.05).

*NE* nonencapsulated, *SPI* soybean protein isolate, *LCC* lecithin calcium caseinate, *GA* gum arabic, *MD* maltodextrin, *RS* resistant starch.

### Effects of wall materials on the morphologies of spray-dried bitter gourd powders

**Fig. 2:**
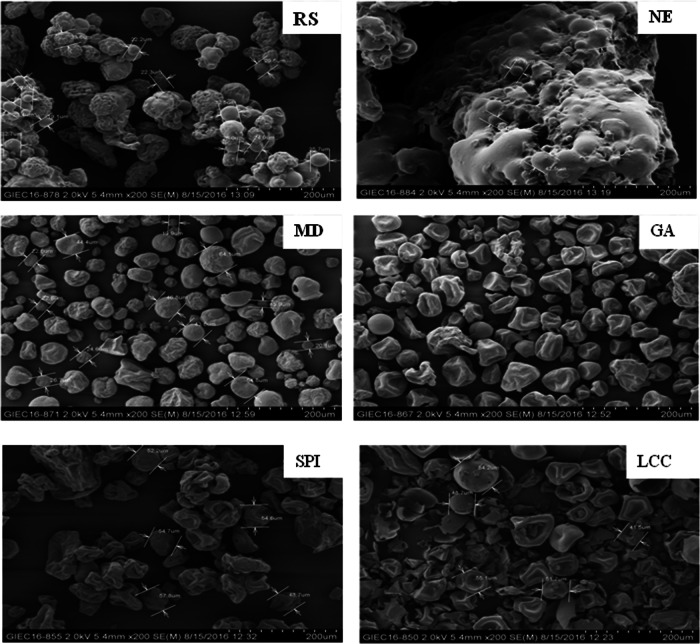
Effects of wall materials on the morphologies of the dried bitter gourd powder particles studied using scanning electron microscopy at 200× magnification. NE nonencapsulated, SPI soybean protein isolate, LCC lecithin calcium caseinate, GA gum arabic, MD maltodextrin, and RS resistant starch.

Figure 2 shows the morphologies of the bitter gourd powder particles observed by scanning electron microscopy. The results indicate that the particles of the NE SD-BGP were mainly globular with a serious phenomenon of aggregation. The addition of the five wall materials decreased particle aggregation compared with that without encapsulation, resulting in perfect encapsulation with few fractures and collapses. The particles of the SD-BGPs prepared with MD and RS were mainly globular, with slight aggregation occurring in the case of RS. The particles of the SD-BGP prepared with GA were mainly umbilicated or globular. In contrast, those prepared with SPI and LCC were umbilicated and irregular in shape, with unique wrinkled surfaces characteristic of protein-based wall materials and larger particle sizes than those of other SD-BGPs prepared with the other three wall materials. Therefore, different wall materials had different effects on the morphologies of the SD-BGP and exhibited quite distinctive surface characteristics. The morphologies of powders, in turn, affect product physicochemical properties; for example, the wrinkled surfaces of SD-BGP may contribute to a delay in bitterness release when they are consumed^46^.

### Effects of wall materials on the particle size distribution of the spray-dried bitter gourd powders

**Fig. 3:**
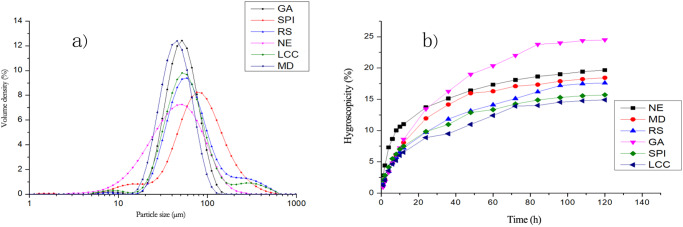
Particle size distribution and hygroscopicity curves of the bitter gourd powders prepared with different wall materials. **a** Particle size distribution and **b** hygroscopicity curves. NE nonencapsulated, SPI soybean protein isolate, LCC lecithin calcium caseinate, GA gum arabic, MD maltodextrin, and RS resistant starch.

Figure 3a and Table 4 display the particle size distribution of the SD-BGPs prepared with different wall materials. The results reveal significant differences in particle sizes between the wall materials, with D^3,4^ ranging from 50.43 to 102.86 μm and the span ranging from 1.06 to 2.27. The NE SD-BGP particles were small in size, had a large span, and presented an irregular particle shape. In contrast, the particles of the SD-BGP prepared with proteins and RS had a larger span and size, with SPI showing the largest D^3,4^ and RS showing the largest span. However, the particles of the SD-BGP prepared with MD and GA were smaller but had more even particle sizes. Generally, small particles have a relatively large surface area per unit mass, and particles with even surfaces have more contact points between particles^47^; this may be the reason for the poor dispersibility of GA- and MD-encapsulated SD-BGP, as shown in Table 2. Conversely, the SD-BGPs prepared with SPI, LCC and RS had larger and more uneven particles, leading to fewer contact points per unit mass. This may be the reason for their good dispersibility, as shown in Table 2.

**Table 4:** Particle size distribution analysis of the bitter gourd powders prepared with different wall materials

Wall materials	Particle size analysis	
	Span	D[3,4] (μm)
NE	1.89	56.27
SPI	1.88	102.86
LCC	1.76	82.57
GA	1.07	58.30
MD	1.06	50.43
RS	2.27	93.92

Different lowercase letters in a column indicate significant differences (*p* < 0.05).

*NE* nonencapsulated, *SPI* soybean protein isolate, *LCC* lecithin calcium caseinate, *GA* gum arabic, *MD* maltodextrin, and *RS* resistant starch.

### Effects of wall materials on the hygroscopicity of spray-dried bitter gourd powders

The results of the hygroscopicity test (Fig. 3b) showed that LCC, SPI, MD and RS decreased the hygroscopicity of the SD-BGP compared with that of the NE SD-BGP, with the greatest reduction achieved with protein-based wall materials, especially LCC. However, the application of GA decreased the hygroscopicity of the SD-BGP only in the first 24 h. Afterwards, the hygroscopicity significantly increased, leading to a much greater hygroscopicity than that of the NE SD-BGP in the end. The higher hygroscopicity of GA-encapsulated SD-BGP than of MD-encapsulated SD-BGP in this study was in agreement with the findings of Ramakrishnan et al., who reported that the hygroscopicity of GA-encapsulated tamarillo juice powder was much greater than that of MD-encapsulated powder^48^. The differences in hygroscopicity among these different wall materials are probably caused by the differences in the chemical structures of the wall materials. GA is a complex heteropolysaccharide with a ramified structure and more hydrophilic groups, which results in a greater water binding capacity^48^.

### Effects of wall materials on the TDA bitterness and sensory effects of spray-dried bitter gourd powders

**Fig. 4:**
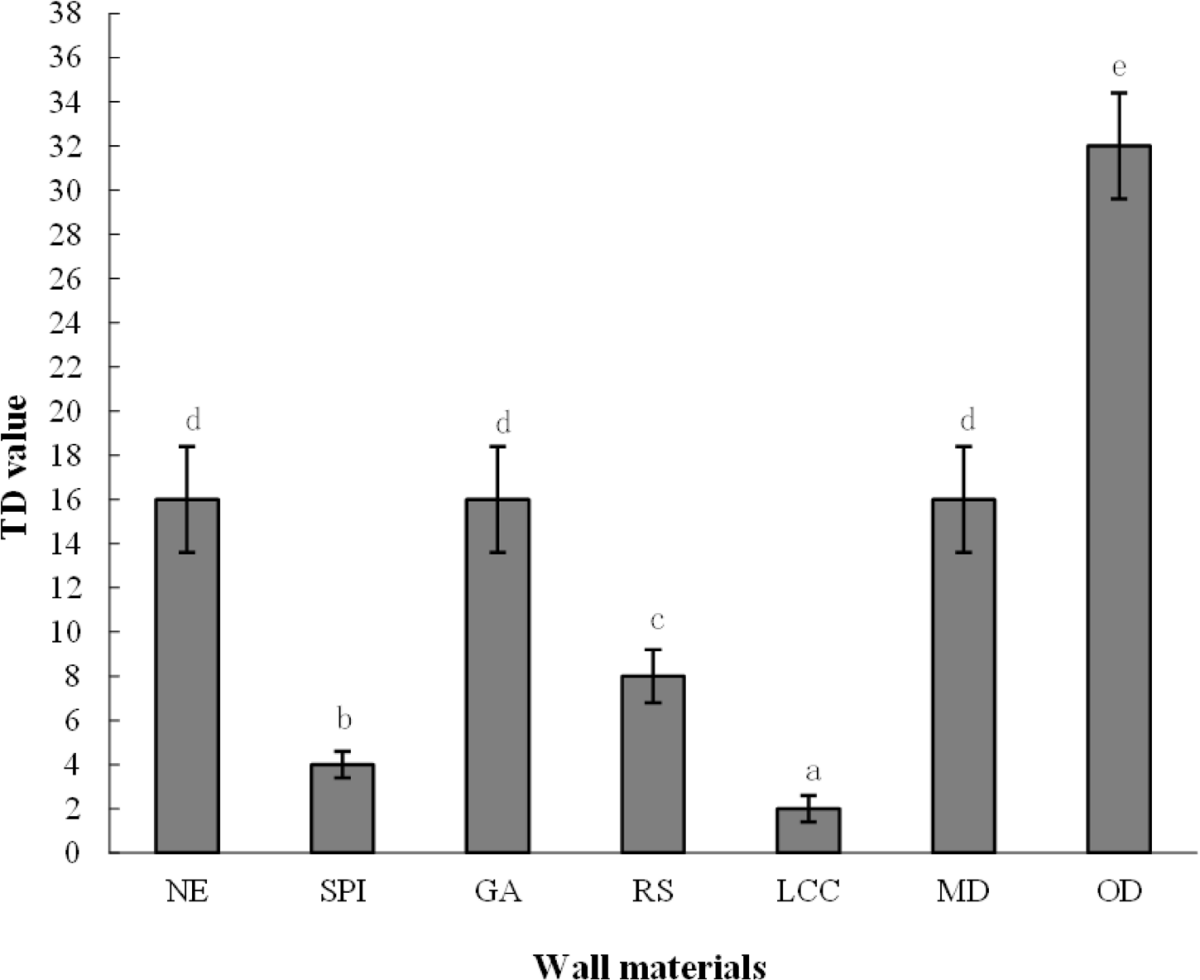
Effects of wall materials on the bitterness of spray-dried bitter gourd powders (±SD). Different lowercase letters indicate significant differences (*p* < 0.05). NE nonencapsulated, SPI soybean protein isolate, LCC lecithin calcium caseinate, GA gum arabic, MD maltodextrin, RS resistant starch, and OD oven-dried.

Figure 4 shows the taste dilution (TD) values of different bitter gourd powder samples obtained through taste dilution analysis (TDA). To better understand the effect of wall materials on the bitterness of SD-BGP, oven-dried bitter gourd powder (OD-BGP) was used as a control. The results revealed that the OD-BGP had the highest TD value, indicating extreme bitterness. In contrast, the NE SD-BGP had a much lower TD but still had a strong bitterness. Among the wall materials, protein-based materials had a significant effect on reducing TD values, with LCC showing the most pronounced ability to mask bitterness, with a TD value of 2, which is eight times lower than that of the NE SD-BGP. The SPI exhibited a TD value of 4, four times lower than that of the NE SD-BGP. RS was also effective in reducing the TD, with a value of 8, while GA and MD had no effect on lowering the TD. The low TD values observed for LCC and SPI may be related to the fact that the elastic and resilient protein films were formed on the particle surfaces, and the particles were umbilicated and irregular in shape with unique wrinkled surfaces and large particle sizes. All these factors prevent the release of bitter substances in water to some extent. The relatively high TD values of GA and MD may be caused by the high solubility of GA and MD in water, thus leading to more bitter substances being released in water.

### Effects of wall materials on the bitterness of spray-dried bitter gourd powders measured using an electronic tongue

**Fig. 5:**
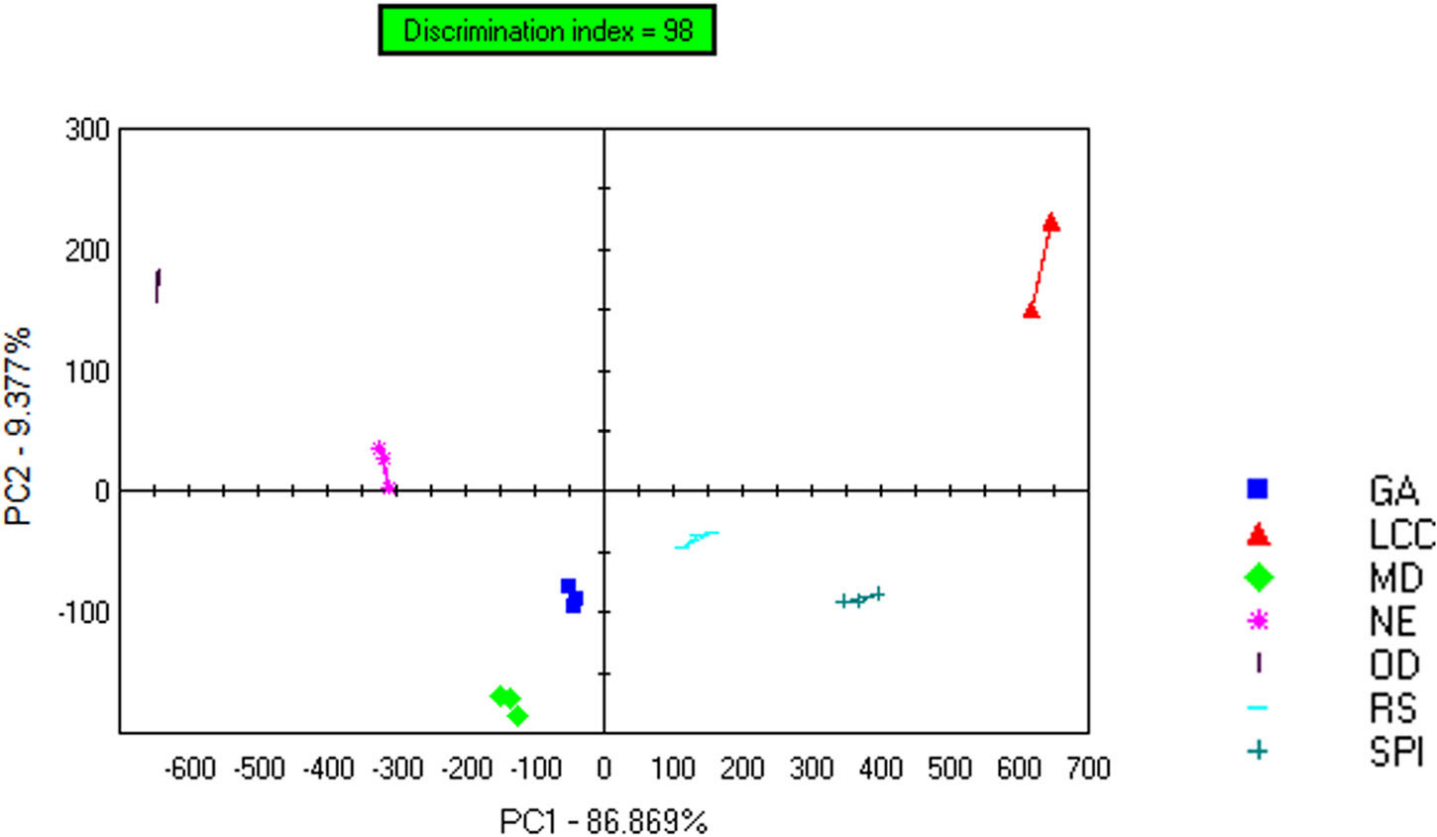
PCA of the tastes of the bitter gourd powders measured with an electronic tongue. NE nonencapsulated, SPI soybean protein isolate, LCC lecithin calcium caseinate, GA gum arabic, MD maltodextrin, RS resistant starch, and OD oven-dried.

Figure 5 presents the results of the principal component analysis (PCA) of the bitter gourd powders based on tastes measured with an electronic tongue. The first two principal components (PC 1 and PC 2) accounted for 96.24% of the combined contribution, indicating that they can effectively reveal the taste profiles of the samples. Additionally, the high pattern discrimination index of 98 suggested that the seven samples could be satisfactorily distinguished from one another based on their taste profiles.

Table 5 displays the distances between the bitter gourd powder samples based on PCA. The closer the distance between samples is, the more similar their tastes are. The shortest distance was observed between GA and MD, while the farthest distance was observed between LCC and OD. The greater the distance between groups and the smaller the distance within a group simultaneously, the closer the pattern discrimination index between two groups is to 100%. All the distances between the SD-BGP and OD-BGP were greater than 400, indicating a significant difference in taste between the two groups, with the addition of wall materials influencing the taste profile.

**Table 5:** Distance between the bitter gourd powder samples

Product names	Reference samples	Distances	Pattern discrimination index (%)
GA	MD	158.23	95.03
GA	RS	225.54	97.04
RS	SPI	281.61	97.55
GA	NE	324.54	98.80
MD	NE	338.58	98.84
MD	RS	365.05	98.80
LCC	SPI	394.28	97.07
NE	OD	407.66	99.42
GA	SPI	419.79	99.05
NE	RS	472.87	99.29
MD	SPI	520.00	99.36
LCC	RS	576.22	98.64
MD	OD	615.78	99.74
GA	OD	658.74	99.79
NE	SPI	713.13	99.66
GA	LCC	740.61	99.23
OD	RS	824.38	99.82
LCC	MD	862.85	99.42
LCC	NE	982.88	99.56
OD	SPI	1049.48	99.88
LCC	OD	1283.80	99.76

*NE* nonencapsulated, *SPI* soybean protein isolate, *LCC* lecithin calcium caseinate, *GA* gum arabic, *MD* maltodextrin, *RS* resistant starch, and *OD* oven-dried.

Table 6 presents the relative intensity of different tastes, including bitterness, sourness, sweetness, umami and saltiness, of various bitter gourd powder samples as measured by the fifth sensor of the electronic tongue. The results indicate that there were notable differences in taste among the different samples. All SD-BGP samples had lower bitterness than the OD-BGP samples, and the addition of wall materials to the SD-BGP samples resulted in lower bitterness than that of the NE SD-BGP samples. The intensity of bitterness in ascending order was LCC, SPI, RS, GA, MD, NE and OD powders. This trend was generally consistent with the pattern observed in the bitterness sensory evaluation (Fig. 4). Among the five wall materials, LCC exhibited the most significant ability to reduce bitterness, with bitterness-masking ratios of 70.53 and 61.64% compared with those of the OD-BGP and NE SD-BGP, respectively. This good bitterness-masking ability of LCC was in agreement with the findings of Hoang Thi et al., who reported that a mixture of lecithin and calcium caseinate had good bitterness-masking efficiency for acetaminophen^39^. SPI also exhibited a significant ability to reduce bitterness, with bitterness-masking ratios of 56.84 and 43.84% compared with those of OD-BGP and NE SD-BGP, respectively. The good bitterness-masking ability of SPI was also confirmed by Molina Ortiz et al., who concluded that spray drying with SPI was an efficient method for microencapsulation and attenuation of the bitter taste of the casein hydrolysate^32^. Compared with those of the OD-BGP and the NE SD-BGP, the bitterness-masking ratios of GA powders were 35.79 and 16.44%, respectively. However, compared with that of the NE SD-BGP, the bitterness-masking ratio of 16.44% in the GA-encapsulated SD-BGP measured in this electronic tongue test was somewhat different from the result observed in the TDA bitterness test, which showed that the TD value of the GA-encapsulated SD-BGP was not significantly different from the TD value of the NE SD-BGP (Table 4). A possible explanation is that the electronic tongue test is more sensitive for bitterness measurements than are human tests. The ability of GA to mask bitterness was also found to be true in hydrolysed casein by Subtil et al., who reported that GA was able to attenuate or mask the bitter taste of hydrolysed casein^27^. Among the five wall materials, MD had the lowest bitterness-masking ability, which may be caused by the rapid and high solubility of MD in water.

**Table 6:** Relative intensity of various tastes of different bitter gourd samples measured with the fifth sensor of the electronic tongue

Sample names	SRS-sourness	GPS	STS_saltiness	UMS-umami	SPS	SWS-sweetness	BRS-bitterness
GA	5.1	6.2	7.1	6.8	5.0	5.3	6.1
LCC	3.8	3.3	1.5	5.2	7.6	5.8	2.8
MD	5.3	6.7	7.7	9.2	6.2	5.1	6.6
NE	9.3	7.5	7.8	3.6	4.6	8.6	7.3
OD	8.9	9.6	6.5	3.9	2.7	9.2	9.5
RS	5.2	4.7	6.3	5.0	7.1	4.6	5.6
SPI	4.5	4.1	5.2	8.2	8.7	3.4	4.1

*NE* nonencapsulated, *SPI* soybean protein isolate, *LCC* lecithin calcium caseinate, *GA* gum arabic, *MD* maltodextrin, *RS* resistant starch, and OD oven-dried.

**Fig. 6:**
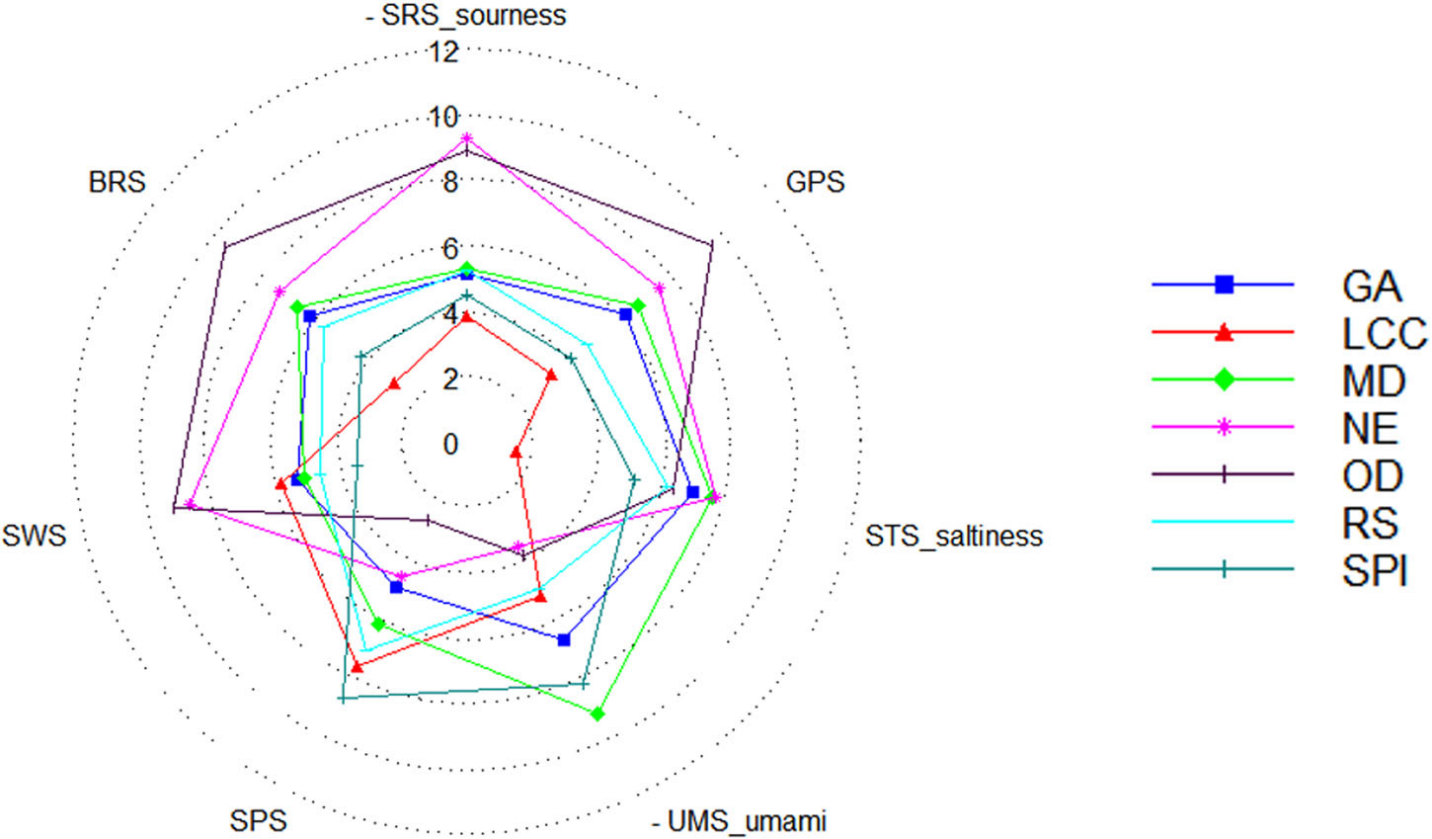
Taste radar graph of different bitter gourd samples. NE nonencapsulated, SPI soybean protein isolate, LCC lecithin calcium caseinate, GA gum arabic, MD maltodextrin, RS resistant starch, and OD oven-dried.

Figure 6 presents a taste radar graph of different samples, which shows the relative intensities of various tastes, ranging from 0 to 12. The graph highlights the differences in taste among the samples, with LCC exhibiting the best result for reducing bitterness.

### Effects of wall material encapsulation on the retention ratio of total phenols

**Fig. 7:**
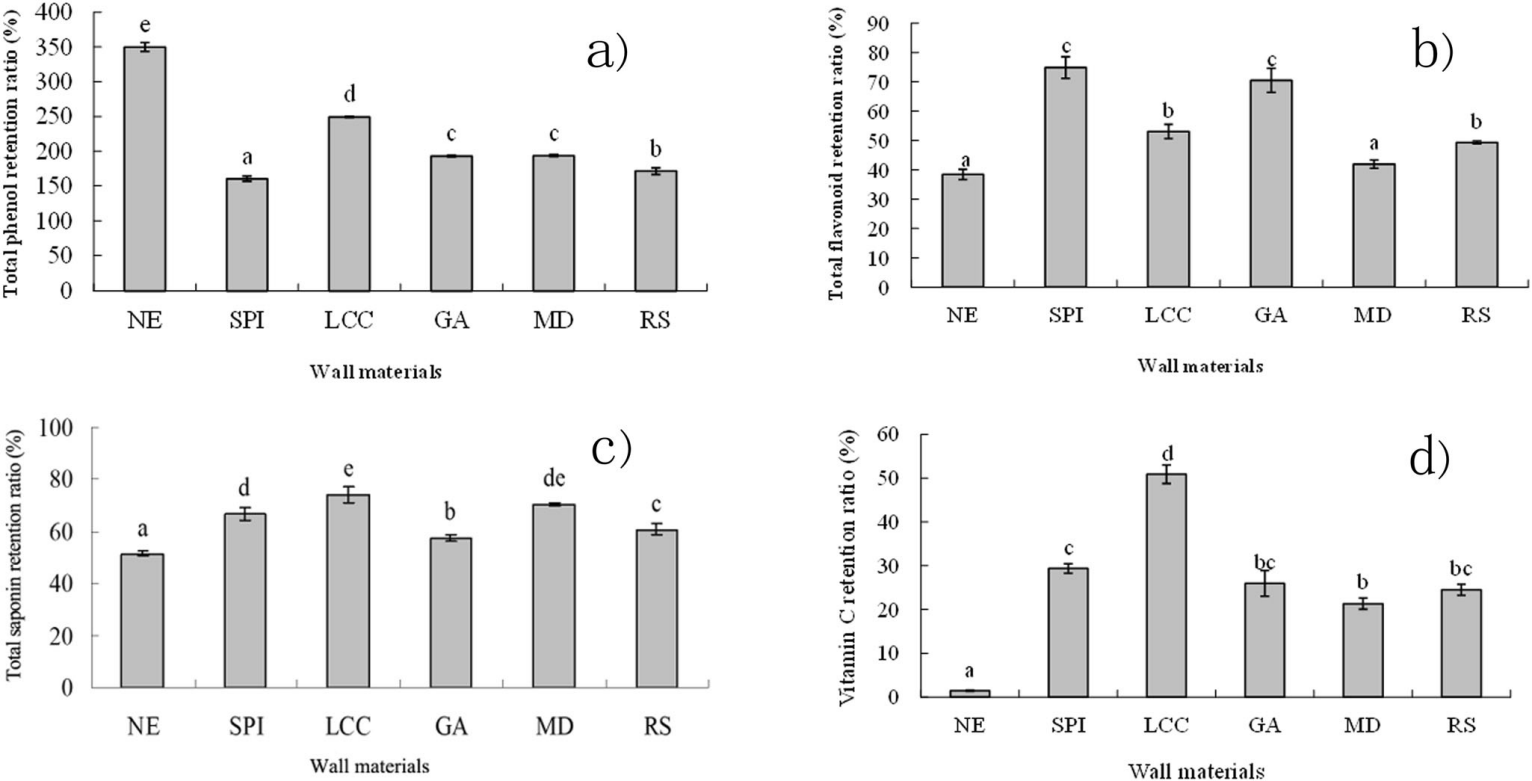
Retention ratios of bioactive components before and after spray drying with different wall materials (±SD). **a** Total phenol retention ratio; **b** Total flavonoid retention ratio; **c** Total saponin retention ratio; and **d** Vitamin C retention ratio. Different lowercase letters indicate significant differences (*p* < 0.05). NE nonencapsulated, SPI soybean protein isolate, LCC lecithin calcium caseinate, GA gum arabic, MD maltodextrin, and RS resistant starch.

Figure 7a presents the retention ratios of total phenols in bitter gourd powders before and after spray drying. The results indicated that spray drying increased the total phenol content, with the NE SD-BGP exhibiting the greatest increase of 346.00%. The total phenol content of the SD-BGPs prepared with various wall materials also increased, with a range of 160.2–249.76%, which was significantly lower than that of the NE SD-BGP. This result was different from the findings of Tan et al. (2015b), who reported that the retention ratios of total phenols in spray-dried bitter gourd extract powders decreased when MD/GA was used as a wall material (below 100%). The reason for this phenomenon may be the difference in the core materials used; a bitter gourd slurry was used in this study, while a bitter gourd extract was used in the study conducted by ref. ^41^. Among the samples prepared with different wall materials, the retention ratio in the case of SPI was the lowest, followed by that of RS. The retention ratios of GA and MD were similar and not significantly different. Notably, LCC exhibited the highest retention ratio at 249.76%, which was significantly greater than the retention ratios observed in samples prepared with the other four wall materials.

### Effects of wall material encapsulation on the retention ratio of total flavonoids

Figure 7b shows the retention ratios of total flavonoids before and after spray drying. The results showed that the percentage of total flavonoids retained after spray drying was less than 80%, indicating that spray drying decreased the total flavonoid content. A retention ratio of 38.99% was observed for the NE SD-BGP, while retention ratios ranging from 42.78 to 75.33% were measured for those prepared with various wall materials, which were higher than those of the NE SD-BGP, indicating that encapsulation with these wall materials was conducive to the retention of total flavonoids. Among the samples prepared with various wall materials, the highest retention was achieved by GA and SPI, with retention ratios of 71.37 and 75.53%, respectively, with no significant difference between them. This was followed by RS and LCC, with retention ratios of 49.01 and 53.11%, respectively, both of which were significantly greater than that of the NE SD-BGP. The retention ratio of total flavonoids by MD was the lowest, which was not significantly different from that of the NE SD-BGP. The relatively high retention ratio of total flavonoids by GA and low retention ratio by MD observed in the present study were consistent with the results of Tan et al., who compared the effect of different ratios of MD to GA on the retention of total flavonoids in encapsulated bitter melon powders and discovered that a greater ratio of GA in combination with MD led to a greater retention ratio^41^, indicating that GA had a greater effect on the retention of total flavonoids than MD. The results of this study are also supported by Ramakrishnan et al., who used MD, GA and three other wall materials to encapsulate tamarillo juice through spray drying and discovered that GA had the highest retention of total flavonoids among these five wall materials, while MD had the lowest retention^48^.

### Effects of wall material encapsulation on the retention ratio of total saponins

Figure 7c shows the retention ratios of total saponins before and after spray drying. The results indicated that spray drying caused a decrease in the total saponin content, with a retention ratio of 52.21% observed for the NE SD-BGP and retention ratios ranging from 57.68 to 74.00% for samples prepared with various wall materials, which were significantly greater than those of the NE SD-BGP. Among the samples prepared with various wall materials, GA had the lowest retention ratio, followed by RS, with retention ratios of 57.68 and 61.13%, respectively, both of which were still significantly greater than that of the NE SD-BGP. The retention ratios of MD and SPI were 70.38 and 65.83%, respectively, which were not significantly different. The retention ratio for LCC was the highest among the samples prepared with wall materials, at 74.00%. However, the relatively low retention ratio of total saponins by GA and high retention ratio by MD observed in this study were inconsistent with the results of Tan et al., who compared the effect of different ratios of MD to GA on the retention of total saponin content of encapsulated bitter melon powders and discovered that a greater ratio of GA in combination with MD led to a greater retention ratio of total saponins^41^, indicating that GA had a greater effect on the retention of total saponins than MD, which is not supported by our observation. A possible explanation is the difference in the core materials used; a bitter gourd slurry was used in this study, while a bitter gourd extract was used in the study conducted by ref. ^41^.

### Effects of wall material encapsulation on the retention ratio of vitamin C

Figure 7d shows the retention ratios of vitamin C before and after the spray-drying process. The findings revealed that the vitamin C content decreased during spray drying, with the NE SD-BGP showing a retention ratio of 1.28%, indicating that almost all the vitamin C was lost. However, the retention ratios for samples prepared with various wall materials ranged from 57.68 to 74.00%, which were significantly greater than those of the NE SD-BGP. Among the various wall materials, MD exhibited the lowest retention ratio of 20.97%, followed by GA, RS and SPI, with retention ratios of 26.85, 24.48 and 29.17%, respectively, with no significant differences among them. In contrast, the retention ratio of LCC was 51.69%, which was significantly greater than that of the SD-BGP samples prepared with the other four wall materials. The high temperature used for spray drying can easily degrade vitamin C, as confirmed by the very low retention ratio of vitamin C in the NE SD-BGP, as shown in Fig. 7d. When the five wall materials were used in spray drying, the retention ratio of vitamin C became much greater, indicating that all these wall materials may have a protective effect on vitamin C via encapsulation, thus preventing direct exposure of vitamin C to high temperature.

### Effect of wall material encapsulation on the oxygen radical absorbance capacity

**Fig. 8:**
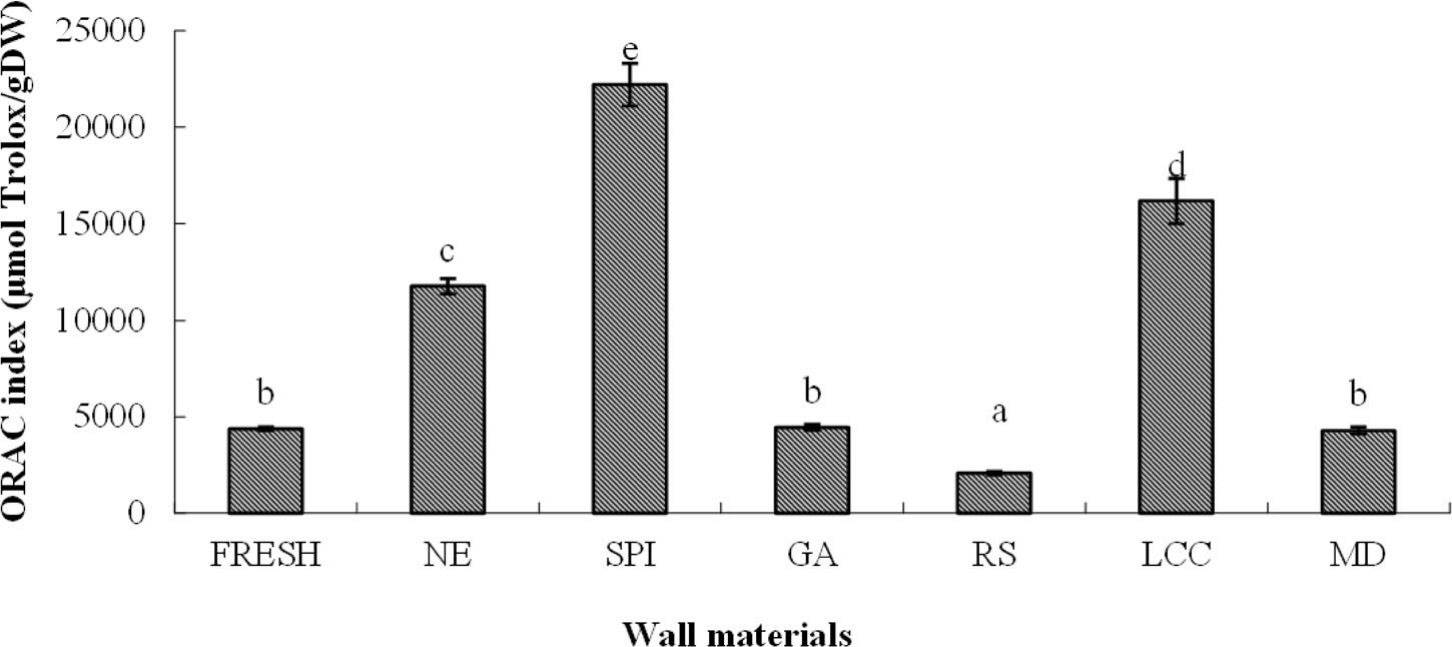
ORAC indices of various samples (±SD). Different lowercase letters indicate significant differences (*p* < 0.05). NE nonencapsulated, SPI soybean protein isolate, LCC lecithin calcium caseinate, GA gum arabic, MD maltodextrin, RS resistant starch, and Fresh fresh bitter gourd.

The findings presented in Fig. 8 demonstrate that various wall materials had differential effects on the ORAC indices of the SD-BGP. The ORAC index of the fresh bitter gourd was 4348.78 μmol TE/g DW, whereas the ORAC index of the NE SD-BGP substantially increased to 11746.15 μmol TE/g DW. Notably, the ORAC indices of the powders prepared with protein-based wall materials significantly increased, with the highest ORAC index of 22209.76 μmol TE/g DW observed for the powder prepared with SPI. In contrast, the ORAC index of the powder prepared with RS decreased significantly, with an ORAC index of 2075.19 μmol TE/g DW. The ORAC indices of the powders prepared with MD and GA were not significantly different from those of the fresh bitter gourd. The ORAC indices were ranked in ascending order as follows: RS, fresh bitter gourd, GA, MD, NE, LCC and SPI. Proteins can keep bioactive compounds from being destroyed due to their good encapsulation capacity in spray drying, thus increasing the retention ratio of these bioactive compounds, which may be the reason that protein-based wall materials such as SPI and LCC generally have higher retention ratios of these bioactive compounds (Fig. 7), leading to higher ORAC indices in the cases of SPI and LCC (Fig. 8). The antioxidant activity of the SPI and LCC themselves may also be a contributing factor to the higher ORAC indices in the cases of the SPI and LCC in this study.

### Effects of wall material encapsulation on the inhibitory activity against α-glucosidase

**Fig. 9:**
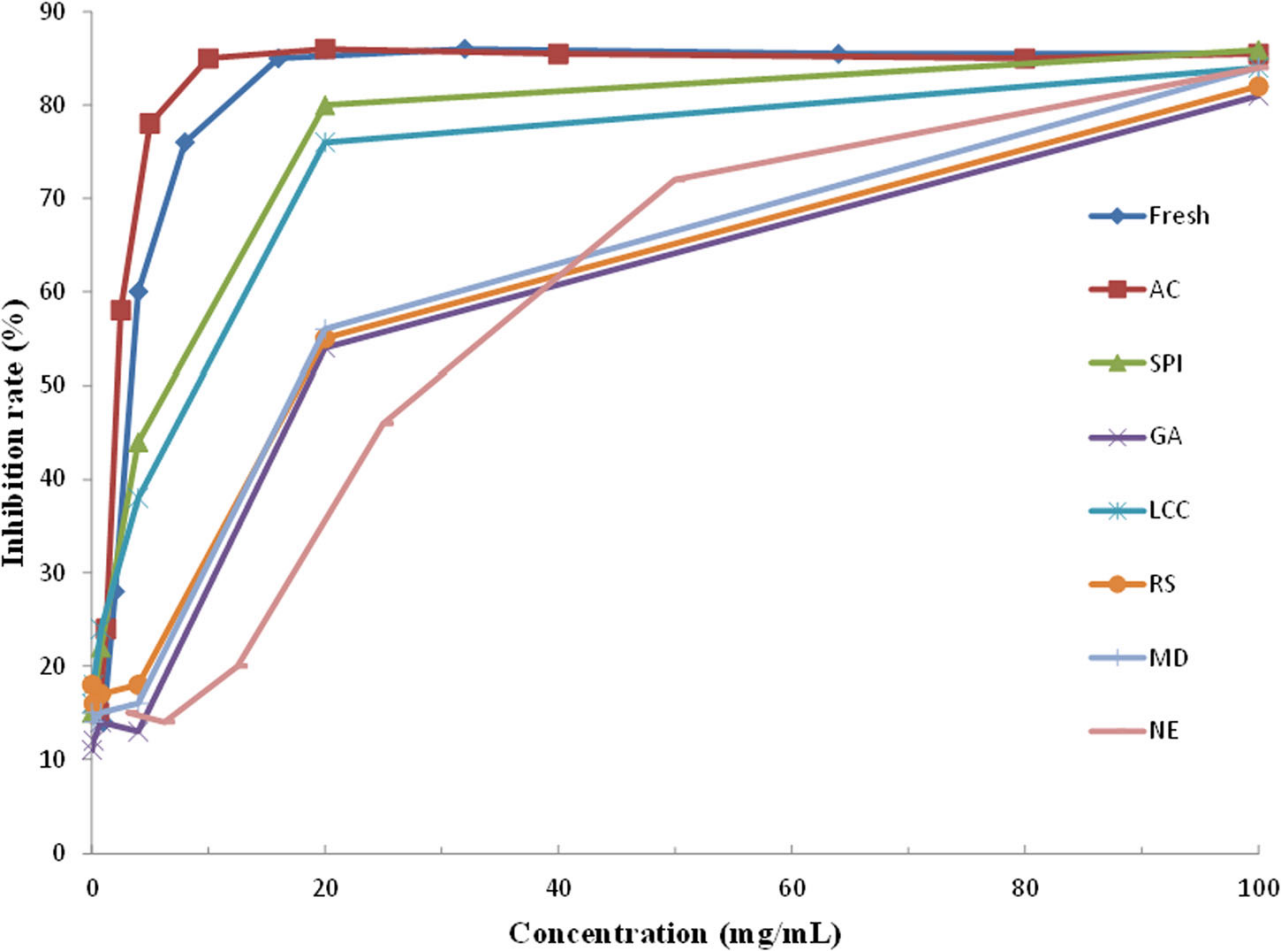
α-Glucosidase activities of various samples. NE nonencapsulated, SPI soybean protein isolate, LCC lecithin calcium caseinate, GA gum arabic, MD maltodextrin, RS resistant starch, Fresh fresh bitter gourd, and AC acarbose.

Figure 9 and Table 7 present the inhibitory activity against α-glucosidase of various samples, and the results demonstrated that wall material encapsulation significantly affected the inhibitory activity against α-glucosidase. The inhibitory activity of all SD-BGP samples against α-glucosidase was lower than that of fresh bitter gourd. NE SD-BGP had the lowest inhibitory activity against α-glucosidase, with an IC_50_ value of 27.56 mg/mL. However, among the samples prepared with various wall materials, the powder prepared with SPI exhibited the highest inhibitory activity against α-glucosidase, with an IC_50_ of 5.20 mg/mL. This value was relatively close to the IC_50_ of the control acarbose (2.14 mg/mL), indicating satisfactory inhibitory activity against α-glucosidase. The IC_50_ values of the SD-BGP samples in ascending order were SPI (5.20 mg/mL), LCC (8.73 mg/mL), MD (17.43 mg/mL), GA (17.46 mg/mL), RS (17.82 mg/mL) and NE (27.56 mg/mL). This ascending order based on the IC_50_ values roughly conforms to the descending order based on the retention ratio of total saponins (Fig. 7c), indicating that the inhibitory activities of SD-BGPs against α-glucosidase are closely related to the saponin content in the powders.

**Table 7:** The inhibition ratios for α-glucosidase

Sample	IC_50_ (mg/mL)
AC	2.14
FRESH	3.08
NE	27.56
SPI	5.20
GA	17.46
RS	17.82
LCC	8.73
MD	17.43

*NE* nonencapsulated, *SPI* soybean protein isolate, *LCC* lecithin calcium caseinate, *GA* gum arabic, *MD* maltodextrin, *RS* resistant starch, *Fresh* fresh bitter gourd, and AC acarbose.

## Discussion

According to refs. ^49,50^, when the moisture content is less than 6% and the water activity is less than 0.3, it is possible to inhibit biochemical and microbial reactions in the food system, which can further prevent oxidative deterioration of the product. The addition of different wall materials caused a decrease in moisture content in this study, possibly due to the rapid migration of the wall materials to the water‒air interface and the fast formation of a film on the particle surface, promoting the quick movement of water molecules^38,45^. The lower moisture content and water activity of SD-BGP achieved by encapsulating with SPI, LCC, GA and MD in the spray-drying process will be beneficial for extending the shelf life of the products.

Reconstitution of a given powder represents four process steps: wetting of particles, sinking of particles below the liquid surface, dispersion of particles in the liquid and complete dissolution of particles into the liquid; each of these processing steps can be related to the properties of the powders (wettability, sinkability, dispersibility and solubility)^51^. The incorporation of MD and GA in bitter gourd powders was found to enhance their solubility due to the high water solubility of these two wall materials^43^. Moreover, smaller particle sizes are known to improve the solubility. SPI, LCC, and RS, which have larger particle sizes, were found to have poor solubility. The powders prepared with GA and MD are more difficult to disperse in water than other wall materials, probably due to their smaller particle size^51^ and lower moisture content. As a wall material, MD could negatively affect powder dispersion in SD-BGP, and this finding was also confirmed by Baldelli et al., who reported that MD increased dispersion time in water in milk powders^44^. Previous studies have suggested that a higher moisture content leads to larger particle sizes and improved instant solubility. However, this characteristic may also be influenced by other factors, such as the polarity of the particle materials^52^.

The NE SD-BGP, which was produced through spray drying, had a dark red colour and lacked the distinctive green colour of fresh bitter gourd. This colour change may be attributed to the oxidation of pigments at high inlet air temperatures and higher sugar contents during the spray-drying process, as reported in previous studies^33,34^. However, the addition of various wall materials with excellent film-forming properties helped prevent oxidation of the core material, thus preserving the green colour of the bitter gourd to some extent. The increase in the yellowness and whiteness values may be attributed to the colour of the wall materials themselves. Furthermore, compared with those of powders prepared with other wall materials, the surface morphology of the SD-BGP prepared with resistant starch appeared to be spherical and in an aggregated state. The aggregation of particles is likely caused by the increase in viscosity resulting from their lower glass transition temperature, as reported in previous studies^38,45^. The addition of high-molecular-weight GA, MD, SPI and LCC can significantly increase the glass transition temperature of powders, thereby preventing particle agglomeration. Furthermore, proteins can migrate to the particle surface and quickly form films, overcoming particle-to-particle adhesion during spray drying^38,45^. Using protein as a wall material results in a protein-specific wrinkled surface, with gaps on the powder surface caused by uneven shrinkage during the drying process. The plasticity of the protein film formed on the surface of the droplet increases, allowing the particles to shrink without breaking the film, leading to shrunken and rough particle surfaces. The irregular morphologies such as dimples and folds on the protein surface may be due to the lower inlet air temperature and film-forming properties of the wall materials^53^. The folded surface morphology provides a smaller contact area between particles than does a spherical surface, leading to less attraction between particles and reducing the possibility of interparticle adhesion^54^. The absence of obvious cracks on the surface of the SD-BGP indicates that the core material is well embedded in the wall material. This suggests that the microcapsules have low gas permeability, increasing the retention ratio of active ingredients and enhancing their protective effect^55^.

Small particles are typically preferred for addition to a food system because they ensure uniformity and quality, and the presence of large particle-size ingredients is generally undesirable in most food products^51,56^. However, larger particle sizes may have a better protective effect on the core material than small particle sizes, as smaller particle size powders have a larger surface area per unit mass. This larger surface area could increase powder adhesion by providing more accessible points for adhesion and interaction between particles^47^. The differences in the particle size distributions may be attributed to the film-forming ability and colloid properties of the various wall materials.

The bitterness of the SD-BGP was lower than that of the OD-BGP. There are several reasons for the reduction in bitterness. First, during the spray-drying process, the content of saponin, the main substance responsible for bitterness in bitter gourd, decreased. Second, encapsulation of different wall materials can have a slow-release effect on bitter substances^39^. The presence of different wall materials can slow the movement of bitter substances to bitter taste receptors, and hydrophobic interactions between wall materials and bitter substances can reduce exposure to bitter substances^57^. Additionally, the intensity of bitterness may be related to the solubility of the powder in water, and a decrease in solubility leads to a decrease in exposure to hydrophobic bitter substances^27^.

The SD-BGP with LCC as the wall material had the greatest ability to mask bitterness. The possible reason for this is that during the spray-drying process, proteins tend to aggregate at the air-liquid interface of droplets because of their surface-active properties. The reorganisation of adsorbed molecular structures during the adsorption process may lower the exposure of hydrophobic groups towards the air interface^58^. Additionally, the film-forming ability of proteins decreases hygroscopicity and swelling, thereby delaying the release of substances^39^. The presence of calcium ions in calcium caseinate may promote intermolecular forces through the interaction of calcium ions with carboxyl groups or other anions^59^. The addition of soybean lecithin is also conducive to the delayed release of bitter substances. Both proteins and small-molecule surfactants can adsorb to the interface through different mechanisms, forming a stronger shell, so the powder prepared with LCC has the lowest bitterness^60^. RS has a relatively strong bitterness-masking ability, possibly due to its lower solubility, resulting in less exposure to bitter substances.

The addition of wall material to bitter gourd leads to an increase in viscosity and surface-to-volume ratio, which can effectively protect the functional components from high-temperature degradation or damage from other harmful factors. As a result, the various functional components of bitter gourd can be better retained during the spray-drying process^61^. In this study, the retention ratios of total flavonoids, total saponins and vitamin C in all the SD-BGPs encapsulated with the wall materials were greater than those in the nonencapsulated SD-BGPs, while the retention ratios of total phenols in all the SD-BGPs encapsulated with the wall materials were lower than those in the nonencapsulated SD-BGPs. The greater increase in polyphenol content in the NE SD-BGP than in the SD-BGP may be due to the bound phenol in the NE SD-BGP not being encapsulated in the wall material. This allows the free phenol to be released more fully.

Proteins exert a good protective effect on total flavonoids, total saponins, vitamin C, and other functional components, possibly due to their strong ability to form films with functional components. This results in good encapsulation and protection of functional components^53,60^. In this study, all spray-dried powders had some antioxidant activity because they retained certain antioxidant components, such as polyphenols, flavonoids, saponins, and vitamin C^62^. Research by Tan et al. (2015b) showed that bitter gourd powders encapsulated with MD and GA had better retention ratios of total phenols, total flavonoids, and total saponins. Antioxidant activity was correlated with the total phenolic content, total flavonoid content, and total saponin content to a certain extent^41^. The SD-BGPs encapsulated with SPI and LCC exhibited the highest antioxidant activity. This may be attributed to the high retention ratios of total phenols, total flavonoids, total saponins, and total vitamin C in the SD-BGPs encapsulated by SPI and LCC and to the antioxidant activity of these wall materials^63,64^. All SD-BGPs have some α-glucosidase inhibitory activity mainly because they contain a certain amount of saponins, which are widely recognised for their α-glucosidase inhibitory activity^9^.

In summary, SPI, GA, MD, RS and LCC can all help reduce the moisture content, water activity, and degree of browning while improving the water solubility of SD-BGPs. Most wall materials, except for RS, can also retain some degree of green colour in the SD-BGP. All five wall materials can alleviate the agglomeration phenomenon, but they affect the particle size and surface morphology differently. Except for GA, all wall materials can reduce the hygroscopicity of the SD-BGP. Additionally, all five wall materials can help reduce the bitterness of the SD-BGP, with protein-based wall materials exhibiting better bitterness-masking ability and LCC showing the best overall bitterness-masking ability. Spray drying can promote the release of polyphenols from whole bitter gourd fruit, resulting in increased polyphenol content in the bitter gourd powders after spray drying. However, spray drying can also reduce the total flavonoid, saponin, and vitamin C contents. All five wall materials have some protective ability on these compounds, with protein-based wall materials showing the strongest overall protective ability. Specifically, LCC had the strongest protective effect on total flavonoids and total vitamin C, while SPI had the strongest protective effect on total saponins. The SD-BGP exhibited some antioxidant and hypoglycaemic activity, with protein-based wall materials showing good overall results. Specifically, the SPI showed the best results. The discoveries in this study may be applicable to producing similar spray-dried vegetable or fruit powders. The most suitable wall material for a specific physicochemical property can be chosen based on the findings of this study depending on the purpose of spray drying, and the best results can be achieved by further optimisation of the spray-drying conditions.

## Materials and methods

### Materials

Fresh bitter gourd of the variety ‘Lvbaoshi’ was obtained from the Tianpingjia Fruit Wholesale Market in Tianhe District, Guangzhou Municipality, China.

### Reagents and instruments

The ginsenoside Rg1 reference substance was purchased from Chengdu Must Biotechnology Co., Ltd. (Chengdu, China). Trolox (6-hydroxy-2,5,7,8-tetramethylchroman-2-carboxylic acid), α-glucosidase, catechin, *p*-nitrophenyl-α-d-glucopyranoside (PNPG), 2,2’-azobis (2-methylpropionamidine) dihydrochloride (AAPH), fluorescein disodium salt (FL), and acarbose were purchased from Sigma Aldrich Company (Shanghai, China). Acetone, methanol, Folin phenol, gallic acid, sodium hydroxide, oxalic acid, boric acid, o-phenylenediamine, ascorbic acid, sodium acetate, sodium nitrite, aluminium trichloride, sodium carbonate, perchloric acid, vanilla aldehyde, and glacial acetic acid were all analytically pure. SPI (JL7501, Suihua Jinlong Oil Industry Co., Ltd., Suihua, China), GA (Cangzhou Kangxin Beeswax Colloid Industry Co., Ltd., Cangzhou, China), MD (Shandong Xiwang Sugar Industry Co., Ltd., Binzhou, China), resistant starch (Hefei Tanrun Biotechnology Co., Ltd., Hefei, China), soybean lecithin (HXY-3SP, Tianjin Hexiyuan Phospholipid Technology Co., Ltd., Tianjin, China), and calcium caseinate mixture (Henan Huachi Biotechnology Co., Ltd., Zhengzhou, China) were of food-grade materials. An LCC was produced by mixing soybean lecithin and calcium caseinate at a ratio of 1.5:1.0 and homogenising with a high-speed homogeniser.

A high-speed universal crusher was purchased from Tianjin Taisite Instrument Co., Ltd. (Tianjin, China), an IKA T25 high-speed homogeniser was obtained from IKA (Germany), an EYELA N-1100 rotary evaporator was purchased from Tokyo Rikakikai Co., Ltd. (Tokyo, Japan), and a TD-6 refrigerated centrifuge was purchased from Changsha Xiangzhi Centrifuge Equipment Co., Ltd. (Changsha, China). A Genie multipurpose vortex mixer was obtained from Scientific Industries, Inc. (Bohemia, NY, USA), an Infinite M200pro multimode microplate reader was purchased from TECAN Trading AG (Switzerland), an UltraScan VIS colorimeter was obtained from HunterLab (Virginia, the United States), and a UV-1800 UV‒vis spectrophotometer was obtained from Shimadzu Corp. (Kyoto, Japan). A LabSwift-aw water activity measuring apparatus was purchased from Novasina (Switzerland), an SU-70 thermal field emission scanning electron microscope was purchased from Hitachi Ltd. (Japan), and an MS 3000 laser particle size analyser was obtained from Malvern Panalytical (London, UK). A DHG-9425A electric heating constant-temperature drying oven was obtained from Shanghai Yiheng Technology Co., Ltd. (Shanghai, China), Astree Electronic Tongue was obtained from Alpha MOS (Toulouse, France), an LPG-5 spray dryer was purchased from Changzhou Jinqiu Drying Equipment Co., Ltd. (Changzhou, China), and an AUX936 beater was obtained from AUX Co., Ltd. (Ningbo, China).

### Preparation of spray-dried bitter gourd powders

#### Selection of wall materials

The criteria for selecting a wall material are mainly based on physicochemical properties such as solubility, molecular weight, glass/melting transition, crystallinity, diffusibility, film forming and emulsifying properties and cost^65^. To reduce the bitter taste of bitter gourd powder, five wall materials were selected, namely, SPI, GA, MD, resistant starch (RS), and LCC. The selection of these five wall materials for spray drying was based on previous research and preliminary experiments^1^. SPI is an abundant, inexpensive and renewable raw material that has many useful functional properties for encapsulation^32^. Molina Ortiz et al. used SPI (at concentrations of 8.4 and 9.6% w/w) to encapsulate casein hydrolysate through spray drying and successfully attenuated the bitter taste of the hydrolysate^32^. Favaro-Trindade et al. also used SPI (concentrations ranging from 5.6 to 9.6% w/v) to reduce the bitter taste of casein hydrolysate^2,30^. GA is widely used as an encapsulating material in spray drying because of its good emulsifying capacity and low viscosity in aqueous solution^66^. Subtil et al. found that GA (concentrations ranging from 21-37% w/v) could be used to effectively reduce the bitter taste of casein hydrolysate during spray drying^27^. Cano-Chauca et al. (2005) used GA (at a concentration of 12% w/v) as a wall material to obtain mango powder by spray drying^3,29^. MD is a common drying aid for spray drying owing to its beneficial role as an encapsulating agent and because MD is inexpensive and has high solubility, neutral flavour, and low viscosity at high concentrations^34^. Singh Oberoi et al. used MD (3, 5, 7 and 10%) to obtain watermelon juice powder^33^. Ferraz et al. found that 15% MD in combination with 5% whey protein isolate produced the best protection of the active compounds during storage in spray-dried paprika and cinnamon oleoresin powders^4,35^. RS is a form of modified starch that resists degradation by enzymes^67^. Muhammad et al. (2017) reported that 5% potato-resistant starch provided better results for thermal denaturation and survival of probiotic bacteria in spray-dried powders during storage^5,68^. LCC is a mixture of lecithin and calcium caseinate. Lecithin, which is a mixture of surface-active agents called phospholipids (an anionic surfactant of low-molecular weight), is considered a generally recognised as safe (GRAS) substance^39^. Caseins are phosphorylated proteins, and commercialised caseins are commonly found in the form of calcium or sodium caseinate and are commonly considered to be GRASs.^39^. Sánchez et al. found that proteins and low-molecular-weight surfactants both absorb to the interface by different mechanisms^60^. Therefore, the behaviour of a mixture containing these components is important for determining the surface structure and bulk composition of dried droplets^39^. Hoang Thi et al. reported that a mixture of lecithin and calcium caseinate (at a ratio of 1.5 to 1.0) offered good bitterness-masking efficacy for a drug^39^.

#### Preparation of feed liquid

The fresh bitter gourd fruit was washed, seeded, cut into pieces, and crushed with a high-speed universal crusher for 90 s to form a slurry. The five wall materials (SPI, GA, MD, RS and LCC) were mixed with the bitter gourd slurry at a ratio of 1:1 based on their respective solid contents. The total solid content of the feed liquid was adjusted to 9%. The feed liquid was first beaten with an AUX936 beater at 14,000 revolutions per minute (rpm) for 15 min under ice bath conditions and then homogenised with an IKA T25 high-speed homogeniser at 30 MPa for 5 min.

#### Spray drying

The spray drying conditions were selected according to a previously developed method in our laboratory. The prepared feed liquid was pumped into an LPG-5 spray dryer for spray drying under the following conditions: an inlet air temperature of 130 °C, an atomisation speed of 100 rpm, and a feeding speed of 2 rpm. The resulting spray-dried powder was collected, sealed in a bag, and stored at −20 °C for later use.

### Determination of moisture content

Approximately 0.5 g of a sample (M1) was weighed, placed in a weighing bottle (M2) that was predried to a constant weight, and dried to a constant weight in a DHG-9425A electric heating constant-temperature drying oven at 105 °C. The weight of the weighing bottle with the dried sample (M3) was recorded^69^. Each experiment was repeated three times. The moisture content was calculated as follows:

1 $$X=\left(1-\frac{{\rm{M}}3-{\rm{M}}2}{{\rm{M}}1}\right)\times 100 \% ,$$

where *X* indicates the moisture content in the sample (%), M1 indicates the weight of a sample (g), M2 indicates the weight of the weighing bottle (g), and M3 indicates the weight of the weighing bottle with the sample after drying completed (g).

### Determination of water activity

Two grams of spray-dried powder was used as a sample to measure water activity with a Novasina LabSwift-aw apparatus (Novasina, Switzerland). The temperature and duration for testing were set to 25 °C and 30 min, respectively. Each dried sample was analysed in triplicate, and the average of these three replicates was calculated and used as the water activity for that sample.

### Determination of water-soluble index (WSI)

Approximately 2.5 g of a sample was accurately weighed (dry weight W0), dissolved in 30 mL of water, and mixed vigorously under vortexing conditions for 1 min in a 50 mL centrifuge tube. After 10 min, the sample was mixed again under vortexing conditions for 1 min. The sample was centrifuged at 5000 rpm for 10 min, and the supernatant was transferred to a weighing bottle (weight of the weighing bottle: W1). It was then dried at 105 °C to a constant weight, and the dry weight W2 was measured and recorded^41,70^. The water-soluble index (WSI) was calculated as follows:

2 $$Y=\frac{{\rm{W}}2-{\rm{W}}1}{{\rm{W}}0}* 100 \% ,$$

where *Y* indicates the water-soluble index in the sample (%), W0 indicates the weight of a sample (g), W1 indicates the weight of the weighing bottle (g), and W2 indicates the weight of the weighing bottle with sample after drying was completed (g).

### Determination of the instant solubility

Fifty micrograms of the sample was accurately weighed and placed in a 2 mL centrifuge tube. The sample was dissolved in 1 mL of deionized water and mixed at half speed at room temperature under vortexing conditions. The time (s) needed for complete reconstitution was recorded. Each sample was analysed in triplicate^71^.

### Colour difference analysis

An UltraScan VIS colorimeter was used for direct measurement. After calibration with black and white tiles, colour was measured in reflection macropore mode, and L*, a* and b* values were recorded. The colour intensity (C) and hue angle (H°) were used as measuring indicators^72^. Each sample was measured in triplicate. The L* value represents the lightness and darkness of the colour, the a* value represents the degree of red–green colour, and the b* value represents the degree of yellow–blue colour. The powder was also photographed with a Canon G11.

3 $${\rm{H}}^{\circ} ={\rm{arctg}}({{\rm{b}}}^{* }/{{\rm{a}}}^{* }),{\rm{for}}\; {\rm{a}}* > 0{\rm{;}}\; {\rm{b}}* \,>\, 0,$$

4 $${\rm{H}}^{\circ} \,=\,180^{\circ} +{\rm{arctg}}({{\rm{b}}}^{* }/{{\rm{a}}}^{* }),{\rm{for}}\,{{\rm{a}}}^{* }\, < \,0{\rm{;}}\,{{\rm{b}}}^{* }\, > \,0,$$

5 $${\rm{H}}^{\circ}\, =\,270^{\circ} +{\rm{arctg}}({{\rm{b}}}^{* }/{{\rm{a}}}^{* }),{\rm{for}}\,{{\rm{a}}}^{* }\, < \,0{\rm{;}}\,{{\rm{b}}}^{* }\, < \,0,$$

6 $${\rm{H}}^{\circ} \,=\,360^{\circ} +{\rm{arctg}}({{\rm{b}}}^{* }/{{\rm{a}}}^{* }),{\rm{for}}\,{{\rm{a}}}^{* }\, > \,0{\rm{;}}\,{{\rm{b}}}^{* }\, > \,0,$$

7 $${\rm{C}}={[{({{\rm{a}}}^{* })}^{2}+{({{\rm{b}}}^{* })}^{2}]}^{1/2},$$

8 $${\rm{H}}^\circ ={\tan }^{-1}({\rm{b}}/{\rm{a}}).$$

### Morphological analysis

Scanning electron microscopy (SEM) was used for morphological analysis^73^. The morphology of the spray-dried powders was evaluated with a Hitachi SU-70 thermal field emission scanning electron microscope. A small amount of sample was taken and placed on conductive tape. Gold was sprayed on the surface, and the working voltage was set to 2 kV. The observation was conducted at a magnification of 200×.

### Particle size analysis

The average particle size and particle size distribution of the bitter gourd powder particles were determined using the dry method with an MS 3000 laser particle size analyser. The refractive index and absorptivity were set to 1.52 and 0.1, respectively. The average particle size was expressed as the volume-weighted average Volume D $[\mathrm{4,3}]$, and the particle size distribution was expressed as the span factor^74–76^:

9 $${\rm{Span}}=[{{\rm{d}}}_{[{\rm{v}},90]}-{{\rm{d}}}_{[{\rm{v}},10]}/{{\rm{d}}}_{[{\rm{v}},50]}].$$

### Hygroscopicity analysis

An appropriate amount of bitter gourd powder was placed in a silicon pentoxide desiccator to dry to a constant weight. Then, 5 g of bitter gourd powder was accurately weighed and placed in a weighing bottle, after which its total weight was measured. The weighing bottle was then placed in a glass desiccator with a supersaturated solution of sodium chloride with a relative humidity of 75% and kept in a 25 °C incubator. The weight was measured at 1, 2, 4, 6, 8, 10, 12, 24, 48, 60, 72, 84, 96, 108 and 120 h. The percentage (%) of moisture absorption was calculated for each time point, and a hygroscopicity curve was plotted by plotting the time against the percentage (%) of moisture absorption^77^.

### Determination of the TD value of taste dilution analysis

Bitterness was evaluated following the taste dilution analysis (TDA) method of ref. ^78^. Samples of 50 mg/mL wall material-encapsulated spray-dried bitter gourd powder, 25 mg/mL oven-dried bitter gourd powder, and 25 mg/mL nonencapsulated spray-dried bitter gourd powder were prepared with distilled water. The samples were gradually diluted at a ratio of 1:1, and the bitterness was evaluated using the triangular test. The TD value refers to the dilution factor at which a difference in taste between the diluted and blank samples was detected.

### Electronic tongue analysis

Half a gram of the wall material-encapsulated spray-dried bitter gourd powder, 0.25 g of the oven-dried bitter gourd powder, and 0.25 g of the nonencapsulated spray-dried bitter gourd powder were weighed accurately, and 10 mL of distilled water was added to the mixture. The mixture was soaked for 5 min before filtering, and the filtrate was placed in a 25 mL special electronic tongue beaker for testing. The experiments were conducted using the Astree Electronic Tongue system.

### Determination of total polyphenol content

The total polyphenol content was determined according to the procedures described by Garjani et al. ^79^ and Nawirska-Olszanska et al. ^80^ with some modifications. Briefly, an appropriate concentration was prepared by diluting the polyphenol extract, which had been extracted with 80% acetone and brought to volume with methanol. Subsequently, the sample solution (0.125 mL) and blank control methanol (0.125 mL) were mixed well with 0.5 mL of distilled water and 0.125 mL of Folin phenol reagent, and 1 mL of distilled water and 1.25 mL of 7% (m/v) Na_2_CO_3_ solution were added. The mixture was allowed to stand for 90 min at room temperature in the dark, and the absorbance was measured with a UV-1800 UV‒vis spectrophotometer at 760 nm. Each sample was measured in triplicate. The total polyphenol retention ratio was calculated according to the following equation:

10 $${Total\; phenol\; retention\; ratio}=\frac{{\rm{Total\; phenol\; content\; before\; spray\; drying}}-{\rm{Total\; phenol\; content\; after\; spray\; drying}}}{{\rm{Total\; phenol\; content\; before\; spray\; drying}}}\times 100 \% .$$

### Determination of the total flavonoid content

The total flavonoid content was determined according to the procedures described by ref. ^74^ and ref. ^81^ with some modifications. Briefly, the polyphenol extract that was brought to volume with methanol was diluted to a suitable concentration. The sample solution (0.3 mL) and blank control methanol (0.3 mL) were removed, and 1.5 mL of distilled water and 0.09 mL of 5% (m/v) NaNO_2_ solution were added, mixed separately, and allowed to react at room temperature for 6 min. Afterwards, 0.18 mL of 10% (m/v) AlCl_3_ ∙ 6H_2_O solution was added to the mixture, and after standing for 5 min, 0.6 mL of 1 mol/L NaOH solution was added to the mixture. The resulting solution was brought to 3 mL with distilled water, and the absorbance at a wavelength of 510 nm was measured. Each sample was analysed in triplicate. The total flavonoid retention ratio was calculated according to the following equation:

11 $$\begin{array}{l}{Total\; flavonoid\; retention\; ratio}=\\\frac{{\rm{Total}}\; {\rm{flavonoid}}\; {\rm{content}}\; {\rm{before}}\; {\rm{spray}}\; {\rm{drying}}-{\rm{Total}}\; {\rm{flavonoid}}\; {\rm{content}}\; {\rm{after}}\; {\rm{spray}}\; {\rm{drying}}}{{\rm{Total}}\; {\rm{flavonoid}}\; {\rm{content}}\; {\rm{before}}\; {\rm{spray}}\; {\rm{drying}}}\times 100 \% .\end{array}$$

### Determination of the total saponin content

The total saponin content was determined according to the procedures described by refs. ^82,83^ with some modifications. Briefly, saponin was extracted from bitter gourd powders with ethanol at room temperature under the action of ultrasound. The sample was vacuum filtered, evaporated with a rotary evaporator, and brought to the required volume to prepare the saponin methanol solution. A certain volume of saponin methanol solution or methanol (blank control) was added to a stoppered test tube and evaporated to dryness in a water bath at 60 °C. Then, 0.2 mL of 5% vanillin-glacial acetic acid and 0.8 mL of perchloric acid were added, mixed well, and incubated in a water bath at 60 °C for 15 min. The samples were removed and cooled to room temperature under running tap water. Glacial acetic acid (5 mL) was added to the mixture and shaken well. The mixture was allowed to stand at room temperature for 20 min, after which the absorbance was measured at 568 nm. Each sample was analysed in triplicate. The total saponin retention ratio was calculated according to the following equation:

12 $$\begin{array}{l}{Total\; saponin\; retention\; ratio}=\\\frac{{\rm{Total}}\; {\rm{saponin}}\; {\rm{content}}\; {\rm{before}}\; {\rm{spray}}\; {\rm{drying}}\,-\,{\rm{Total}}\; {\rm{saponin}}\; {\rm{content}}\; {\rm{after}}\; {\rm{spray}}\; {\rm{drying}}}{{\rm{Total}}\; {\rm{saponin}}\; {\rm{content}}\; {\rm{before}}\; {\rm{spray}}\; {\rm{drying}}}\times 100 \% .\end{array}$$

### Determination of the total vitamin C content

Three samples of different spray-dried bitter gourd powders (approximately 1 g of each sample) were weighed accurately, 10 mL of 2% oxalic acid was added to the samples, and the samples were ground sufficiently. Treated activated carbon (0.2 g) was added to the mixture and shaken vigorously. The resulting mixture was centrifuged at 5000 r/min for 10 min. Then, 1 mL of the supernatant was pipetted into sample tube A and blank tube B. Subsequently, 1 mL of 250 g/L sodium acetate solution and 1 mL of 30 g/L boric acid-250 g/L sodium acetate solution were added to tubes A and B, respectively. The solutions were mixed well and allowed to stand in the dark for 20 min at room temperature. Next, 1 mL of 0.2 g/L o-phenylenediamine solution was added to each test tube in the dark. The solutions were mixed thoroughly, and the reaction was conducted in the dark for 40 min. Finally, the fluorescence intensity of each tube was measured at a laser wavelength of 355 nm and an emission wavelength of 425 nm. The relative fluorescence intensity of the sample was determined by subtracting the fluorescence intensity of the sample blank tube from the fluorescence intensity of the sample tube (AOAC, 1985)^84^. Triplicate measurements were taken for each sample. The total vitamin C retention ratio was calculated according to the following equation:

13 $$\begin{array}{ll}{Total\; vitamin\; C\; retention\; ratio}=\\\frac{{\rm{Total}}\; {\rm{vitamin}}\; {\rm{C}}\; {\rm{before}}\; {\rm{spray}}\; {\rm{drying}}\,-\,{\rm{Total}}\; {\rm{vitamin}}\; {\rm{C}}\; {\rm{after}}\; {\rm{spray}}\; {\rm{drying}}}{{\rm{Total}}\; {\rm{vitamin}}\; {\rm{C}}\; {\rm{before}}\; {\rm{spray}}\; {\rm{drying}}}\times 100 \% .\end{array}$$

### Determination of the oxygen radical absorbance capacity (ORAC) index of antioxidant activity

With some modifications, the procedures described by ref. ^5^ were used to determine the ORAC index of antioxidant activity. The total phenolic extracts of the spray-dried bitter gourd powders and fresh bitter gourd were diluted to an appropriate concentration with 75 mmol/L phosphate buffer solution (pH 7.4). In each well, 20 μL of Trolox standard solution (standard), 20 μL of phosphate buffer solution (pH 7.4) (blank), and 20 μL of total phenol sample solution at a certain concentration of bitter gourd were added. Next, 200 μL of 0.96 μmol/L FL working solution was added to each well and incubated at 37 °C for 20 min, after which 20 μL of freshly prepared 119 mmol/L AAPH solution was quickly added to each well except the F well. The multifunction microplate reader was started immediately, and the fluorescence intensity decay of each well was continuously measured with an excitation wavelength of 485 nm and an emission wavelength of 538 nm, with one cycle every 4.5 min, for a total of 35 cycles. The ORAC index was expressed as Trolox equivalents per gram of sample on a dry basis (μmol TE/g DW). Each sample was measured in triplicate.

### Measurement of α-glucosidase activity

The methods described by refs. ^85,86^ were adopted with some modifications. Briefly, saponins extracted from spray-dried bitter gourd powder and fresh bitter gourd were diluted with 0.1 mol/L phosphate buffer (pH 6.8). Subsequently, 50 μL of the aforementioned diluted saponin solutions was added to a 96-well plate, followed by the addition of 25 μL of 0.7 U/mL α-glucosidase. The mixture was then incubated at 37 °C for 10 min. Next, 25 μL of 10 mM PNPG was added to the mixture and incubated at 37 °C for 30 min. Following the incubation, 100 μL of 0.2 mol/L Na_2_CO_3_ solution was added to stop the reaction, and the absorbance was measured at 405 nm. The positive control group (acarbose), blank control group, and sample background control group were also measured under the same system. The enzyme activity inhibition ratio was calculated as follows:

14 $$\alpha -{Glucosidase\; activity\; inhibition\; ra}{tio}=\frac{{\rm{Ak}}-({\rm{Ay}}-{\rm{Ab}})}{{\rm{Ak}}}\times 100 \% ,$$

where Ak, Ay and Ab are the absorbances of the blank, sample and background groups, respectively.

### Statistical analysis

The experiments were performed in triplicate, Excel software was used to tabulate the data, and all the data were expressed as the mean ± standard deviation. The data were plotted with Origin software. To evaluate the effects of different wall materials on the moisture content, water activity, WSI, dispersion time, colour, TDA value, retention ratio of bioactive components and ORAC index of spray-dried longan bitter gourd powders, significance tests of the differences in these physicochemical properties were performed via Duncan’s test via one-way ANOVA with SPSS 18.0 software (SPSS Inc., Chicago, IL, USA). A *p* value of 0.05 was considered the significance threshold.

## Data Availability

The data that support the findings of this study are available from the corresponding authors, Z. Wei and M. Zhang, upon request.
